# Predictors of first-year GPA of medical students: a longitudinal study of 1285 matriculates in China

**DOI:** 10.1186/1472-6920-14-87

**Published:** 2014-04-23

**Authors:** Ying-Xue Zhou, Zhi-Tao Zhao, Li Li, Cheng-Song Wan, Cheng-Hua Peng, Jun Yang, Chun-Quan Ou

**Affiliations:** 1 School of Public Health and Tropical Medicine, Southern Medical University, Guangzhou 510515, China; 2 Office of Academic Affairs, Southern Medical University, Guangzhou 510515, China

**Keywords:** National College Entrance Examination, First-year GPA, Medical students, China, Hierarchical linear model

## Abstract

**Background:**

Although medical education has developed rapidly in the last decade, and the National College Entrance Examination (NCEE) is used as the "gold standard" for admission to medical college in mainland China, there is a lack of literature regarding the influence of NCEE score and other factors on the academic performance of medical students. This study aimed to examine potential predictors of first-year grade point average (GPA) for medical students.

**Methods:**

This study included 1,285 students who matriculated at a first-tier medical university in mainland China in 2011. The precollege motivational attitudes for each matriculate were investigated via questionnaire. A hierarchical linear model was fitted to regress first-year GPA on a 100-point scale on NCEE score and other student-level and major-level characteristics.

**Results:**

NCEE score was a significant predictor of both within-major and between-major variation of first-year GPA for medical students. Majors with higher mean NCEE scores had higher mean GPAs, and higher GPAs were observed among those individuals with higher NCEE scores after controlling for major-level characteristics. First-year GPA differed by certain individual socio-demographic variables. Female students had a 2.44-higher GPA on average than did male students. NCEE repeaters had a 1.55-lower GPA than non-repeaters. First-year GPA was associated negatively with parental income but positively with academic self-concept.

**Conclusions:**

NCEE score is an important predictor of the first-year GPA of medical students, but it is not the sole determinant. Individual socio-demographic characteristics and major-level characteristics should be taken into account to understand better and improve the first-year GPA of medical students.

## Background

A student’s first year of college is a vital time for establishing baseline knowledge, positive attitudes, self-confidence, and commitment to studying [1,2], which, in turn, lay the foundation for the student’s subsequent academic success and retention [3]. The first academic year is especially important for medical students, as they are faced with greater difficulties and more stress in their courses and are at higher risk of withdrawal than students with other majors. There is a need to identify specific characteristics that affect the first-year academic performance of medical students. Such studies can help medical faculty and administrations provide better support services to promote student success and increase student retention. Previous studies on predictors of academic performance in medical school are mainly from the US and Europe. Medical education in mainland China has developed dramatically in the last decade. For example, a total of 528,728 students entered medical colleges in 2011, approximately three times as many as in 2000 [4]. Despite the large and rapidly growing number of medical students in China, few studies have focused on Chinese students representing a variety of racial, ethnic, cultural, and socioeconomic backgrounds.

Prior academic achievement is a key predictor of the students’ further achievements at higher levels of study. There have been many investigations in developed countries regarding the predictive power of high school GPA and American College Test (ACT), Scholastic Aptitude Test (SAT), and Medical College Admission Test (MCAT) scores on first-year college GPA [5-7], and the findings of these studies have provided important information for revising the tests in the US and Europe. In mainland China, the National College Entrance Examination (NCEE), known as Gaokao, is a prerequisite for entrance into almost all colleges. Approximately 9.2 million high school graduates took the NCEE in July 2013. However, there is very little empirical evidence of the predictive validity of the NCEE for college academic performance in China. Recently, Bai and Chi [8] found that NCEE total and subject-specific scores can predict undergraduate GPA for all four years in the School of Economics and Management at Tsinghua University. It remains unclear whether NCEE score can predict medical students’ subsequent performance. Based on simple univariate analyses alone, several studies published in Chinese journals have failed to reveal a significant association between NCEE score and academic performance in medical school [9-11]. Further research based on appropriate statistical approaches is required and would inform the selection of the most qualified students for medical education.

To better understand students’ success, it makes sense to consider the backgrounds and other precollege characteristics of newly enrolled students. Some studies have shown that socio-demographic and psychosocial characteristics (e.g., self-beliefs, long-term goals and expectations regarding student-faculty interaction) are important predictors of academic performance in college [2,5,12-18]. However, some other studies have shown that academic success in the first year was not associated with precollege characteristics, including self-concept, psychological state, and aspirations [19,20]. Although previous studies have demonstrated differences in first-year GPA by gender, there are conflicting reports in the literature as to whether male or female students perform better [21-24]. Some researchers stressed that the impact of family socioeconomic status on students’ performance differs largely depending on the economic development level of the region or country [25,26]. The socioeconomic and demographic differences in medical students’ performance require future empirical analysis in more countries and regions.

In addition to individual characteristics, student’s peer environment is regarded as an important predictor of student success [2,27-29]. Quite a few studies have investigated the role of sub-environments (e.g., majors or minors in an institution) in student outcomes [2,27-29]. Our study used a hierarchical linear model to examine student-level and major-level aggregation predictors of first-year GPA, including NCEE score, precollege motivational attitudes, and socio-demographic characteristics, at a first-tier medical university in mainland China.

## Methods

### Participants

In this study, we considered students matriculating in 2011 at Southern Medical University, a first-tier university in mainland China. The participants were enrolled in 28 health-related majors, including Clinical Medicine, Basic Medicine, Chinese Medicine, Preventive Medicine, Stomatology, Nursing, Pharmaceutics, Medical Technology, and Medical Imaging.

### Predictors

Individual information about precollege motivational attitudes and some socio-demographic characteristics were obtained via a freshman survey. Prior to the start of the fall semester of their freshman year, we mailed the questionnaire together with a notice of admission to matriculates in August 2011 and requested that they return the completed questionnaires when registering in September 2011.

We adopted the questionnaire of the Cooperative Institutional Research Program (CIRP) Freshman Survey and translated it into Chinese. The CIRP was proposed by the Higher Education Research Institute in the US and has been widely used in the US and many other regions as a routine instrument for collecting extensive information about entering students before they experience college [30]. The survey covers a wide range of student characteristics, such as students’ expectations, attitudes, values, and goals for college. In the present study, we considered items addressing academic self-concept, authority, social goals, financial goals, and expectation about interactions with faculty. The content validity coding schemes and descriptive statistics of all variables under study are shown in Table 1.

**Table 1:** Coding schemes and descriptive statistics for socio-demographic variables and descriptive statistics for NCEE score and first-year GPA

**Variable**	**Coding scheme**	**N (%)**	**Variable**	**Coding scheme**	**N (%)**
Gender	Female = 1	773 (60.2)	Parental annual	<RMB$5,000 = 1	143 (11.1)
	Male = 0	512 (39.8)	Income†	RMB$5,000-10,000 = 2	257 (20.0)
Ethnicity	Minority = 1	32 (2.5)		RMB$10,000-20,000 = 3	280 (21.8)
	Han = 0	1,253 (97.5)		RMB$20,000-40,000 = 4	260 (20.2)
Residence	Urban = 1	686 (53.4)		RMB$40,000-60,000 = 5	135 (10.5)
	Rural = 0	599 (46.6)		RMB$60,000-80,000 = 6	47 (3.7)
NCEE	Repeater = 1	131 (10.2)		RMB$80,000-100,000 = 7	64 (5.0)
Repetition	Non-repeater = 0	1,154 (89.8)		>RMB$100,000 = 8	62 (4.8)
Parental Education*	First-generation = 1	1,013 (78.8)	NCEE score	Mean (SD)	587.61 (21.79)
	Non-first-generation = 0	272 (21.2)	First-year GPA	Mean (SD)	77.32 (5.30)

*First-generation college students are students from families in which neither parent had more than a high-school education.

†Best estimate of parents’ total income last year (considering income from all sources before taxes) on an eight-point scale.

NCEE score data for each matriculate were provided by the admission and registrar’s office of Southern Medical University. The NCEE is taken by nearly all applicants for university study in China. Senior high school students must choose between science and the liberal arts. Almost all students who want to enter a medical college choose science and then take the NCEE tests for four subjects: mathematics, Chinese, English, and integrated science (including chemistry, physics, and biology). A perfect NCEE score is 750 points, with 150 points for each single-subject test and 300 points for integrated science. Students are allowed to repeat the exam in the following year(s) if their scores due not meet their expectations [31].

### First-year GPA

First-year GPA data were also provided by the admission and registrar’s office. The curricula in the first year of college mainly consist of foundational courses. First-year GPA was calculated using the credit-weighted sum of the grades for all courses divided by the total credits. The grades for each course and GPA were on a 100-point scale.

Each matriculate’s first-year GPA was matched to his/her NCEE score and survey data with the unique examination number used in the NCEE. Among all the 1,820 matriculates in 2011, 1,557 (85%) completed the CIRP freshman survey. Excluding 535 students who did not include their examination number in the questionnaire, a total of 1,285 (71%) students were included in the final analyses. The participants come from 19 provinces, three municipalities, and three autonomous regions; thus, the participants in this study represented 80% (25/31) of the regions in mainland China. The study protocol was approved by the Southern Medical University ethics committee.

### Statistical analysis

#### Univariate analysis

An independent-samples t test was used to determine the differences in the average GPA and NCEE score between groups.

#### Assessment of questionnaire reliability and validity

The internal reliability of the questionnaire was evaluated using Cronbach’s alpha coefficient. To provide conceptual clarity and evaluate construct validity, a principal component factor analysis with varimax rotation was conducted on 16 items related to motivational attitude. Due to the ordinal nature of item responses, both factor analysis and the calculation of Cronbach’s alpha were based on polychoric correlations. Polychoric correlation can produce more accurate estimates of parameters and standard errors than traditional Pearson correlations [32]. All polychoric correlations were computed using the two-step estimation algorithm in the polycor version 0.7-8 package of R version 3.0.2. To minimize the effect of collinearity among factors, we calculated the factor scores using the Anderson-Rubin approach [33].

#### Hierarchical linear modeling analyses

In the present study, we examined major-level and student-level predictors of GPA. Potential student-level (i.e., within-major) predictors included individual NCEE score, five factor scores of precollege motivational attitudes, and socio-demographic characteristics. Major-level variables were measured by the mean or percentage of corresponding student-level variables for all individual students in the same major. The inclusion of group-level variables in an ordinary least squares regression model would lead to the misestimation of standard errors and the use of the incorrect number of degrees of freedom, increasing the likelihood of a Type I error (i.e., indicating that something is statistically significant when it is not) [34]. The hierarchical linear model (HLM) approach allows the intercept to vary by major and the analysis of major-level characteristics, resulting in more accurate parameter estimates than ordinary least square regression. Therefore, we developed an HLM on nested data (individual students nested in majors) to regress GPA on potential predictors of interest. We standardized all continuous and ordinal variables (parental income) to a mean of 0 and standard deviation of 1 before HLM analysis [35,36]. All within-major predictors were group-mean centered, and all between-major predictors were grand-mean centered. The detailed modeling strategy was as follows. We first performed a fully unconditional model, a random-effects analysis of variance (ANOVA), which partitioned the total variation in GPA into within- and between-major components. If the variance of the random effects was statistically significant, indicating that GPA varied significantly between majors, then we built a conditional model including major-level and student-level predictors. If the random coefficients of the predictors were not statistically significant, suggesting that their predictive validity did not vary by major, then we only considered the fixed effects of predictors. To assess the percentage of unique variance explained by the specific sets of variables, we performed a three-step analysis. The following three sets of variables were added in the model in a stepwise manner: major-level variables (Model 1), individual socio-demographic variables (Model 2), and other student-level variables (Model 3). In each step, the index of the proportion reduction in residual variance (δ^2^) was calculated as [δ^2^(unconditional)-δ^2^(conditional)]/δ^2^(unconditional), an equation suggested by Raudenbush and Bryk [37]. The results of the statistical tests were considered to be statistically significant if *p* was less than 0.05, and all statistical tests were two-tailed.

## Results

The results of the univariate analysis, presented in Table 2, revealed that NCEE score differed significantly by ethnicity, residence and NCEE repetition. Females and non-first-generation students had higher GPAs than males and first-generation students (students whose parents did not attend university or college), respectively (*p* < 0.05).

**Table 2:** Comparisons of average GPA and NCEE score between groups

**Variable**	**Variable value**	**N**	**NCEE score**			**First-year GPA**		
			**Mean (SD)**	***t***	***p***	**Mean (SD)**	***t***	***p***
Gender	Female	773	587.51 (22.90)	-0.21	0.836	78.26 (4.73)	7.65	0.000
	Male	512	587.76 (20.00)			75.91 (5.79)		
Ethnicity	Minority	32	554.79 (33.65)	-5.63	0.000	75.74 (5.29)	-1.71	0.087
	Han	1253	588.45 (20.75)			77.36 (5.30)		
Residence	Urban	686	586.10 (22.02)	-2.68	0.007	77.39 (5.46)	0.51	0.612
	Rural	599	589.35 (21.40)			77.24 (5.12)		
NCEE Repetition	Repeater	131	595.92 (25.90)	3.94	0.000	76.51 (5.42)	-1.85	0.065
	Non-repeater	1154	586.67 (21.07)			77.41 (5.28)		
Parental Education	First-generation	1013	587.72 (20.87)	0.30	0.762	77.16 (5.19)	-2.11	0.035
	Non-first-generation	272	587.22 (24.94)			77.92 (5.64)		
Parental annual income	≤ RMB$40,000	940	588.12 (21.25)	-0.98	0.327	77.36 (5.22)	-0.45	0.655
	>RMB$40,000	308	586.72 (23.29)			77.20 (5.58)		

As shown in Table 3, the overall Cronbach’s alpha coefficient was 0.84, and each of the five dimensions had an alpha coefficient higher than 0.70. Principal component factor analysis conducted on the 16 individual items yielded five factors with eigenvalues greater than 1 and cumulatively explained 61.5% of the variance. The factor loadings of all items on the corresponding factor were higher than 0.40 (ranging from 0.49 to 0.99), confirming the postulated structure.

**Table 3:** Factor structure and reliability for motivational attitude factors

**Factors and Survey Items**	**Mean**	**SD**	**Factor**	**Cronbach’s Alpha**
			**Loading**	
**Academic self-concept**				0.82
Academic ability*	3.88	0.60	0.71	
Creativity*	3.42	0.77	0.61	
Drive to achieve*	4.09	0.74	0.63	
Mathematical ability*	3.53	0.85	0.63	
Self-confidence (intellectual)*	3.82	0.76	0.78	
**Authority**				0.74
Becoming an authority in my field^†^	2.79	0.79	0.67	
Obtaining recognition from my colleagues for contributions to my particular field^†^	2.92	0.73	0.68	
Making a theoretical contribution to science^†^	2.73	0.81	0.49	
**Social goals**				0.81
Helping others who are in difficulty^†^	2.99	0.71	0.56	
Participating in a community action program^†^	2.71	0.72	0.66	
Improving my understanding of other countries and cultures^†^	2.64	0.80	0.68	
Adopting "green" practices to protect the environment^†^	2.92	0.79	0.88	
**Financial goals**				0.72
Being able to make more money^‡^	2.29	0.61	0.59	
Being very well off financially^†^	2.30	0.76	0.99	
**Expectation for student-faculty interaction**				0.81
Communicating regularly with professors^§^	3.52	0.59	0.64	
Working on a professor’s research project^§^	3.43	0.62	0.84	
**All items together**				0.84

*Self-ratings of the traits compared with the average person of the same age on a five-point scale: from 1 = lowest 10% to 5 = highest 10%.

^†^Self-ratings of importance on a four-point scale: from 1 = not important to 4 = essential.

^‡^Self-ratings of importance on a three-point scale: from 1 = not important to 3 = very important.

^§^Best guess of future events on a four-point scale: from 1 = no chance to 4 = very good chance.

Table 4 shows the results of the HLM analyses. We did not find any significant two-way interactions between variables (*p* > 0.05, detailed data not shown). According to the results of the final step (Model 3), among all of the major-level variables, majors’ average NCEE score was the only significant predictor of first-year GPA. The students enrolled in majors with higher average NCEE scores would have higher mean GPAs (*b* = 3.04, *p* = 0.022). We also found that first-year GPA was significantly associated with three socio-demographic variables, including gender, parental income, and NCEE repetition, while no significant association was found with parental education or residence. After controlling for other factors, female students had a 2.44-higher average GPA than male students (*b* = 2.44, *p* < 0.001). NCEE repeaters had a 1.55-lower GPA than non-repeaters on average (*b* = -1.55, *p* = 0.001). Parental income was negatively related to students’ grade performance (*b* = -0.30, *p* = 0.049). It was worth noting that the association with the ethnicity was statistically significant in Model 2 but became non-significant after controlling for NCEE score and motivational attitude in Model 3 (*b* = 0.74, *p* = 0.421). After controlling for the effect of major-level and individual socio-demographic characteristics, individual NCEE score (*b* = 1.24, *p* < 0.001) and academic self-concept (*b* = 0.28, *p* = 0.042) were significantly positively related to first-year GPA.

**Table 4:** Students-nested-in-majors HLM analyses for 1,285 matriculates of a medical school in China

	**Fixed effects**^**§**^	**Model 1**^**†**^		**Model 2**^**†**^		**Model 3**^**†**^	
		***b***	***p value***	***b***	***p value***	***b***	***p *** **value**
**Major-level effects**							
	Intercept	77.13*	0.000	77.13*	0.000	77.17*	0.000
*Social-demographic*	% Female	0.04	0.149	0.04	0.133	0.04	0.172
	% Minority	0.04	0.797	0.04	0.808	0.07	0.661
	% Urban	-0.04	0.510	-0.04	0.505	-0.03	0.604
	% NCEE repeaters	0.03	0.675	0.03	0.677	0.04	0.615
	% First-generation	-0.08	0.284	-0.08	0.299	-0.08	0.313
	Average parental income	0.47	0.857	0.40	0.878	0.38	0.888
*Average NCEE*	Average NCEE score	2.96*	0.022	2.91*	0.023	3.04*	0.022
*Motivational attitude*	Average academic self-concept	0.18	0.949	0.41	0.883	0.67	0.815
	Faculty interaction	0.64	0.769	0.87	0.687	1.05	0.638
	Average authority	-1.72	0.381	-2.10	0.287	-2.32	0.252
	Average social goals	3.17	0.212	3.25	0.201	2.93	0.260
	Average financial goals	-1.74	0.491	-1.65	0.513	-1.51	0.559
**Student-level effects**							
*Social-demographic*	Gender: female			2.37*	0.000	2.44*	0.000
	Ethnicity: minority			-1.98*	0.023	0.74	0.421
	Residence: urban			-0.06	0.845	0.09	0.782
	NCEE repeaters			-0.96*	0.035	-1.55*	0.001
	First-generation			-0.23	0.549	-0.27	0.469
	Parental income			-0.33*	0.032	-0.30*	0.049
*Individual NCEE*	NCEE score					1.24*	0.000
*Motivational attitude*	Academic self-concept					0.28*	0.042
	Faculty interaction					0.22	0.106
	Authority					0.05	0.685
	Social goals					0.06	0.666
	Financial goals					0.24	0.071

^†^Model 1 included major-level variables alone; Model 2 included major-level variables and student-level socio-demographic variables; Model 3 included major-level variables and all student-level variables.

*Significant effects (*p* < 0.05) under each of the three models.

^§^*b* is the estimated regression coefficient, *SE* is its standard error, and the statistical magnitude of *p* is based on the *t* ratio.

As shown in Table 5, the random-effects ANOVA (unconditional model) revealed that the majority (85.2%) of the total variation in first-year GPA was due to within-major variation, while the rest (14.8%) was attributable to between-major variation; this result was statistically significant (*Z* = 3.20, *p <* 0.001). Table 5 also presents the variance components of the random effects and proportion reduction in the residual variance for the three conditional models (i.e., hierarchical linear models). The inclusion of all major-level variables in the model (Model 1) led to a reduction of 59.9% and 8.9% in between-major residual variance and total residual variance, respectively, while adding student-level socio-demographic variables in the model (Model 2) reduced the total residual variance by 13.8%. The inclusion of all major-level and student-level variables (Model 3) caused a reduction of 18.2% in the total residual variance, of which 9.3% (i.e., 18.2%-8.9%) was attributable to the student-level variables. We also further broke down the student-level variables to determine how much they explained the total variance and found that 4.9% (i.e., 13.8%-8.9%) was contributed by student-level socio-demographic variables, while 4.4% (i.e., 18.2%-13.8%) was contributed by student-level NCEE score and motivational attitude variables. To quantify the overall contribution of NCEE score to GPA, we removed the two NCEE variables (i.e., majors’ average and student-level NCEE score) from Model 3 and found an increase of 6.8% in total residual variance. Thus, NCEE score explained 6.8% of the variation in GPA.

**Table 5:** Variance component of random effects by ANOVA and proportional reductions in residual variance for conditional models

	**Fully unconditional model**	Conditional model (hierarchical linear model)*					
		**Model 1**		**Model 2**		**Model 3**	
**Random effects**	**Variance component (%)**^**†**^	**Variance component**	**Proportion reduction**^**§**^	**Variance component**	**Proportion reduction**^**§**^	**Variance component**	**Proportion reduction**^**§**^
Between-major residual	4.05 (14.8)	1.63	59.9%	1.63	59.7%	1.76	56.6%
Within-major residual	23.30 (85.2)	23.31	0.0%	21.93	5.9%	20.63	11.5%
Total residual	27.35 (100)	24.93	8.9%	23.57	13.8%	22.39	18.2%

*Conditional model: Model 1 included major-level variables alone; Model 2 included major-level variables and student-level socio-demographic variables; Model 3 included all variables.

^†^"%" denotes the proportion of total variance due to between- and within-major variance, correspondingly.

^§^"Proportion reduction" denotes the proportion reduction in residual variance (δ^2^), calculated by [δ^2^(unconditional)-δ^2^(conditional)]/δ^2^(unconditional).

## Discussion

In the present study, we found that NCEE score was a significant predictor of first-year GPA. In addition, we found that NCEE repeaters performed worse than non-repeaters in medical-based knowledge studies, which was consistent with previous studies in the US [38,39]. Another US study also indicated that scores on the admission test for medical students (i.e., the MCAT) had a positive relationship with GPA in the first two years [40]. One recent analysis has shown that scores on all three versions of the MCAT were moderately correlated to performance measures in medical school in the US [7]. In China, a few previous studies explored the association between NCEE total scores and academic performance in medical school, but none of them observed a significant association, which was most likely due to the use of improper statistical methods [9-11]. These previous studies estimated the effect of NCEE score on students’ achievement using univariate analyses, which did not control for other possible influencing factors. Our study overcomes some shortcomings associated with previous Chinese studies. Firstly, the present study included 1,285 subjects, a much larger sample size than the previous studies [9-11]. Secondly, we considered a number of potential affecting factors of first-year achievement simultaneously, including contextual factors and students’ precollege motivational attitudes. Moreover, the study subjects were admitted to 28 different majors, allowing us to consider major-level effects in the analyses.

There is clear evidence of a gender difference in the first-year GPA, with female medical students outperforming male students. DeBerard et al. [41] suggested two reasons for the gender difference in academic performance in university: degree structures that were more suited to a specific gender and course imbalances in which student populations were predominantly male or predominately female. In a previous US study, Cuddy et al. [21] demonstrated that men tended to outperform women slightly on the USMLE Step 1 examination because more men than women major in basic science disciplines in their undergraduate education. However, shifting from basic to clinical science, in the USMLE Step 2 Clinical Knowledge (CK) examination, women begin to catch up and outperform men [42]. In China, the first-year medical education not only focuses on basic science knowledge but also includes life science courses, such as Medical Cell Biology, Systematic Anatomy, Histology, and Embryology. We found that female students had higher first-year GPAs than male students, most likely because females are generally more interested in studying a science that they perceive as a helping science, a people-oriented science, or a nurturing science [23]. Other explanations are related to gender differences in personality and social psychology. Chinese female students exert more effort in studying than male students because they are more self-disciplined and more inclined to think that good grades are important in landing a good job and therefore care more about their grades.

A few Western studies have shown that family income level and parental education have positive influences on college student academic success [2,43]. In the present study, univariate analysis showed significantly higher GPA for non-first-generation undergraduates than for first-generation undergraduates, while this significant difference disappeared after controlling for other factors in multivariate analysis, suggesting that parental education is not an independent predictor of students’ GPA. However, students’ GPA was negatively associated with parental income. In mainland China, the tuition fee for one school year in medical college is approximately RMB $6,000 (approximately USD $978), much lower than that in the US. Chinese students commonly address their financial problems by seeking financial aid from their families and society rather than by taking on paid employment, as done by Western students. The Chinese government has incrementally introduced various types of financial aid, including scholarships, need-based grants, and loans, to help students pay for college. Moreover, social organizations and universities have also increased aid, ostensibly for disadvantaged college students. As a result, students rarely withdraw because of financial problems. On the contrary, financial disadvantage motivates the students to achieve better grades. They have a stronger aspiration for academic success because they want to change the socio-economic status for themselves and their families more so than other students. They generally spend more time studying after class.

Many studies have shown race differences in academic performance, which was not examined in the present study because all participants were Chinese. In this homogeneous population, ethnic minorities tended to have lower GPAs than ethnic Hans, but the difference was eliminated after controlling for the effect of NCEE score and student precollege attitudes, suggesting that ethnicity is not an independent predictor of GPA. Inconsistent with our result, Bai and Chi [8] found that ethnic minority status had a significant effect on GPA in the first year but not the three subsequent years. It is possible that the percentage of minority students (2.5%) was too small in our study to provide the statistical power to detect small ethnic differences, especially as we considered so many factors simultaneously.

The findings of this study have important practical implications. First and foremost, our findings support the continued use of NCEE score as a predictor of student performance in medical school. However, in the present study, NCEE score explained only 6.8% of the total variance of GPA, comparable to the finding by Bai and Chi [8]. This finding indicates that although NCEE score is an important predictor of GPA, its predictive validity is not high and it is therefore necessary to revise the NCEE. Secondly, we identified several underperforming groups, including males, NCEE repeaters, students whose parents have high income, and students with low academic self-concept. Student affairs practitioners should pay special attention to these lower-performing groups to support them academically. There is evidence that underperforming groups can benefit from specific types of instructional interventions and programs, such as tutoring, supplemental instruction programs, or peer study groups [19]. Students’ beliefs about their academic abilities should be considered when designing instructional experiences in medical education. Thirdly, an enrollment administrative system incorporating major-level NCEE requirement, individual NCEE score, socio-demographic characteristics, and academic self-perception might help improve the selection process.

There are some limitations to this study. Firstly, the completeness, authority, and reliability of the CIRP questionnaire have been verified over decades of studies. We translated the questionnaire into Chinese and found the internal consistency and construct validity to be good. However, we did not assess its test-retest reliability. The questionnaire needs to be improved upon in further Chinese studies. Secondly, all data were obtained from a single medical university. Further research at multiple colleges and universities would enhance the generalizability of the findings. In the present study, we considered only one outcome, first-year GPA, and assessed its association with NCEE score. Further studies are needed to determine whether NCEE score can predict long-term medical school grades, clerkship ratings, and other local indicators of students’ academic performance. Finally, the "outcome", or impact, is the result of student inputs mixing with the environment. Although we considered several major-level factors, there are still some potential affecting factors, such as major policies and activities, social life, and provision of academic support, that may moderate or bias the relationships between GPA and predictors of interest.

## Conclusions

In generalizing the predictors of academic performance to more medical majors, the HLM framework is useful for accounting for the variability due to student-level and aggregate measures of student-level predictors (major-level variables, e.g., percent female students). In the present study, we found that NCEE score is an important predictor of the first-year GPA of medical students, indicating that NCEE, as the entrance examination in China, is a valid evaluation for selecting medical students. On the other hand, NCEE score is not the sole predictor of academic performance. Individual socio-demographic characteristics and major-level characteristics should also be taken into account to better understand and improve students’ academic performance. Interestingly, inconsistent with the findings in the US, we found that female students had higher GPAs than male students and that students whose parents have higher income tended to have lower GPAs. More research in developing countries is required to examine the socioeconomic-demographical differences in medical student performance.

## Abbreviations

NCEE: National College Entrance Examination; GPA: Grade point average; ACT: American College Test; SAT: Scholastic aptitude test; MCAT: Medical College Admission Test; CIRP: Cooperative Institutional Research Program; HLM: Hierarchical linear model.

## Competing interests

The authors declare that they have no competing interests.

## Authors’ contributions

CQ, YX, and ZT initiated the study. ZT, CS, and CH collected and interpreted the data. YX, LL, and JY cleaned up the data and performed the statistical analyses. YX and CQ drafted the manuscript. All authors read and approved the final manuscript.

## Pre-publication history

The pre-publication history for this paper can be accessed here:

http://www.biomedcentral.com/1472-6920/14/87/prepub
